# The Importance of Maize Management on Dung Beetle Communities in Atlantic Forest Fragments

**DOI:** 10.1371/journal.pone.0145000

**Published:** 2015-12-22

**Authors:** Renata Calixto Campos, Malva Isabel Medina Hernández

**Affiliations:** Programa de Pós-Graduação em Ecologia, Departamento de Ecologia e Zoologia, Universidade Federal de Santa Catarina, Florianópolis, Santa Catarina, Brazil; University of Brasilia, BRAZIL

## Abstract

Dung beetle community structures changes due to the effects of destruction, fragmentation, isolation and decrease in tropical forest area, and therefore are considered ecological indicators. In order to assess the influence of type of maize cultivated and associated maize management on dung beetle communities in Atlantic Forest fragments surrounded by conventional and transgenic maize were evaluated 40 Atlantic Forest fragments of different sizes, 20 surrounded by GM maize and 20 surrounded by conventional maize, in February 2013 and 2014 in Southern Brazil. After applying a sampling protocol in each fragment (10 pitfall traps baited with human feces or carrion exposed for 48 h), a total of 3454 individuals from 44 species were captured: 1142 individuals from 38 species in GM maize surrounded fragments, and 2312 from 42 species in conventional maize surrounded fragments. Differences in dung beetle communities were found between GM and conventional maize communities. As expected for fragmented areas, the covariance analysis showed a greater species richness in larger fragments under both conditions; however species richness was greater in fragments surrounded by conventional maize. Dung beetle structure in the forest fragments was explained by environmental variables, fragment area, spatial distance and also type of maize (transgenic or conventional) associated with maize management techniques. In Southern Brazil’s scenario, the use of GM maize combined with associated agricultural management may be accelerating the loss of diversity in Atlantic Forest areas, and consequently, important ecosystem services provided by dung beetles may be lost.

## Introduction

The use of genetically modified (GM) technology in agriculture has increased globally, with the largest increase occurring in Brazil (i.e., an increase of 3.7 million hectares) [1]. The effects of GM plants on non-target organisms are highly controversial. A number of articles have reported no effects (see [2– 4]), while others have described significant negative effects on several invertebrate species [3, 5–13]. A meta-analysis of 42 field experiments concluded that non-target invertebrate groups were less abundant in GM fields compared to insecticide-free fields [2].

The use of GM technology in Brazil is associated with a type of maize management. The manufacturer of GM technology makes available to the farmer, a “technological package" with products and practices that guide this type of culture [14]. Herbicides, for example, are being used in over 70% maize areas in Brazil [15]. Chemical control should be accomplished through the use of herbicides registered and applied in the correct doses. To select an herbicide, the composition of the weeds present, the environmental characteristics in the area to be treated, and the physical and chemical characteristics of the products should be considered [16].

The use of GM crops could mitigate many of the negative effects of insecticides, but insect species that are not susceptible to the expressed toxin can develop into secondary pests and cause significant damage to the crop [8, 17]. Insecticide spraying could become the immediate solution at farmers’ disposal, and the sustainable use of this genetic modification technology may be not occurring [17]. The negative effects of GM crops on associated fauna via trophic webs are poorly understood [5, 18]. Currently, the actions of Bt toxins (extracted from *Bacillus thuringiensis*) are subject to more controversy than when Bt plants were first developed [19]. Transgenic DNA and proteins may pass through mammalian or avian gastrointestinal tracts [20–22], as well as through animals that consume them, where transgenic DNA and proteins circulate in the blood and internal organs [23]. The propagation effect of a disturbance at the trophic level to other levels of the food chain may also be occurring. For example, when honeybees were exposed to a high concentration of Cry1Ab protein (GM maize) the effects were not lethal, but their behavior and learning ability was disrupted [24]. Subtle effects such as aberrations in behavioral or social competence have not been studied to a comparable extent, but these effects may increase or decrease population and community size. The use of some taxon with acknowledged importance in maintaining ecological processes can serve as a tool for finding general patterns related to GM crops cascade effects on wildlife.

A recent study with dung beetles showed changes in functional group dynamics and abundance of some species in communities inhabiting forest fragments surrounded by GM maize [13]. Indirect behavioral effects, for example the search and exploitation of food resources, can generate cascade effects. If a feces provider (mammals) changes their diet, this may have consequences that result in changes to dung beetle communities via trophic cascade effects. Dung beetles (Coleoptera: Scarabaeinae) are extremely important organisms for tropical ecosystem functioning [25] since they promote soil removal and incorporation of organic matter in nutrient cycling, which helps to regulate and improve physical and chemical properties of soil [26–28]. Most species are detritivores, feeding and nesting on feces (coprophagous) or carcasses (necrophagous), both primarily from mammals [29].

Environmental degradation causes changes in dung beetle community structure and composition, resulting in a decrease of species diversity in comparison to preserved areas [30–34]. The dung beetles rapid response to habitat alterations has led to their recognition as efficient ecological indicators [31, 35–39]. In addition to community-level changes, some species show increased or decreased abundance in areas with particular characteristics caused by environmental change, such as communities found in forest fragments surrounded by GM maize, that show an increase of dweller species and a decrease in tunneler species [13]. Changes in habitat complexity modify not only the insect communities, but also the fauna associated with forests, reducing the richness of some taxonomic groups while increasing others [40]. Furthermore, since dung beetle communities depend on mammal excrements, they may be influenced by changes in mammalian assemblages, which are also affected by landscape alterations [41– 42].

The expansion of the agricultural frontier increases fragmentation and subsequently the loss of biodiversity in the Atlantic Forest [43]. In recent studies was found a positive correlation between dung beetle richness and mammal richness and the habitat structure influenced both groups [41–42]. Seventy percent of the Brazilian population lives in the Atlantic Forest, one of the most diverse regions in the world; however these human activities have disturbed this ecosystem [44]. More than 80% of Atlantic Forest fragments are smaller than 50 ha and there is a large average distance between fragments (1440 m) [45]. Dung beetle spatial distribution may be related to geographic distance or lack of connectivity caused by fragmentation [46], and due to limitations in dispersal ability [47–49].

This study was based on the hypothesis that dung beetle communities in forest fragments surrounded by genetically modified maize crops (GM) may be exposed to plant materials and toxins derived from transgenic maize via feces or carcasses of maize-consuming animals and exposed to the maize management techniques of these maize crops (GM). The aim of the present study was to reveal the possible impacts of GMs crops associated with maize management techniques evaluating the type of maize crop (conventional or transgenic), maize management of these crops and others important recognized factors (environmental effects, mammalian presence, and spatial distance) to dung beetle communities in Atlantic Forest fragments surrounded by conventional and transgenic maize.

## Material and Methods

### Study area

The study was conducted in the region of Campos Novos, Santa Catarina state, Southern Brazil (27°23’S, 51°12’W). This region contains several Atlantic Forest fragments, originally Araucaria Forest [50], surrounded primarily by soybean and maize crops. The region has a mild mesothermal climate according to the Köppen classification system with an altitude ranging from 739 to 953 m and distributed rainfall throughout the year, with annual average of 1750 mm approximately [51].

**Fig 1:**
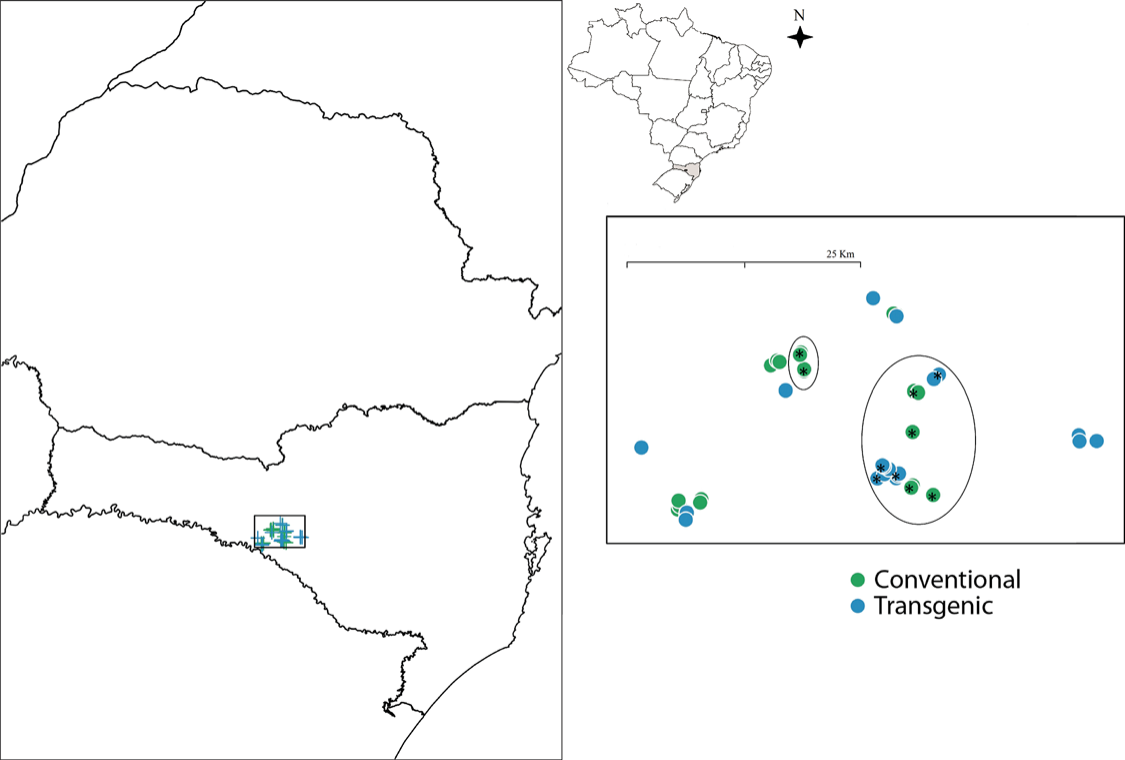
Map of the study region. Location of the 40 forest fragments in the Campos Novos, Santa Catarina state, southern Brazil, near conventional or transgenic maize crops. The twenty fragments sampled in the first year (2013) are circled.

Forty sample areas were established within forest fragments, twenty fragments were surrounded by GM maize crops (ten fragments per year), and twenty fragments were surrounded by conventional maize crops (ten fragments per year) (Fig 1). Farms were chosen with the assistance of the Enterprise for Agricultural Research and Rural Extension of Santa Catarina (Empresa de Pesquisa Agropecuária e Extensão Rural—Epagri/Campos Novos), based on their accessibility and degree of isolation of forest fragments in relation to the type of maize cultivation. Only forest fragments adjacent to monocultures were chosen.

#### Scarabaeinae sampling

Sampling of copro-necrophagous beetles was performed within the forest at a distance of at least 10 m from the fragment’s edge. Each fragment was sampled only once during the study’s two years. Sampling was intensive during the summer, in February 2013 and 2014, during the period right before the maize harvest. Pitfall traps were made using plastic containers (30 cm circumference and 20 cm height), buried in the ground, and protected against rain with plastic cap, lastly, a detergent/water mixture and 10 g of bait were added to each trap. The each sampling point consisted of ten traps per fragment, with 400 pitfall traps across the 40 forest fragments sampled. The fragment was used as the sampling unit. The half of traps was baited of human feces and other half with carrion (pork meat) in order to attract the majority of species (i.e., both coprophagous and necrophagous).

After 48 h of exposure, insects captured were fixed in 70% alcohol and taken to the Laboratory of Terrestrial Animal Ecology (LECOTA/UFSC) for identification [52]. Subsequently the insects were deposited in either the Entomological Collection of the Center for Biological Sciences, Federal University of Santa Catarina (UFSC) or the Entomological Collection of Federal University of Mato Grosso (UFMT). Ten individuals per species were weighed (dry weight) using an analytical balance. To find the total biomass of each fragment, the number of individuals was multiplied by the mean biomass per species. The Instituto Chico Mendes de Conservação da Biodiversidade ICMBio/MMA) issued the permits to collect specimens (permit #32333–3 to MIMH). The field study did not involve any endangered or protected species.

#### Assessment of explanatory variables

Environmental variables–In order to assess the structure of the vegetation in each sampled fragment, an adapted quadrat-section method was used [53]. Using a cross as a reference, four quadrants (northeast, northwest, southeast, southwest) were marked, and in each quadrant the first tree to the center of the cross with diameter at breast height (DBH) greater than 5 cm and the first shrub with a perimeter less than 15 cm and a height greater than 1 m were selected, the following were measured for each tree and shrub: the distances to the center of the cross, the height, and the crown and trunk diameter. Trunk diameter was taken at breast height (1.3 m) for trees and ankle height (DAH = 0.1 m) for shrubs. Furthermore, in each quadrant, height of leaf litter in 1 x 1 m square was measured with a ruler, and percentages of leaf litter layer, green area and exposed soil (no vegetation or leaf litter) were measured by visual estimation using the following classes, 0–5%, 6–25%, 26–50%, 51–75%, 76–95% and 96–100%. Using these same classes, the percentage of canopy cover in the four quadrats was visually estimated, using a square paperboard with a hollow area of 10 x 10 cm, placed at a distance of 40 cm from the observer’s eye, at an inclination of 20° in relation to the zenith. For each variable measured was used an average of eight measurements taken between the pitfalls, in two points in the fragment. The area of each fragment was determined using Google Earth Path (1.4.4a), and altitude using a hand-held GPS.

Management of crop fields—Issues regarding crop management were obtained through semi-structured interviews with nine farmers and four employees, where questions related to agricultural practices used in the properties. The questions were about the maize variety, cattle presence after harvest, use of insecticide, herbicide, and fungicide, and if transgenic crops were ever grown on the site evaluated. All respondents authorized the use of interview data, and interviews lasted on average 15 minutes. Three properties only grew transgenic varieties, eight only grew conventional varieties, and two properties grew conventional and transgenic varieties.

Mammal sampling—Camera traps (BUSHNELL Trophy Cam HD) were used to record mammalian presence inside the forest fragments. Only medium and large mammals were included in the analysis due to the difficulty of identifying little mammal species (rodents) with camera traps. Humans were included because in some fragments the pictures depict hunters, revealing that hunting is present inside the forest. One camera trap was placed in each fragment after dung beetle sampling. Maize from the adjacent crop and meat (to attract predators) were used as bait in front of the cameras. Camera traps were active for a minimum of 40 days and maximum of 60 days. Baits were replaced and batteries checked every 20 days and mammals were identified from the photographs.

Spatial variables—Data from geographic coordinates (Universal Transverse Mercator) obtained at each fragment using a hand-held GPS were used to create spatial variables.

#### Data analysis

The Jackknife 1, Chao 1 and Chao 2 estimators were used to estimate dung beetle richness in sampled fragments, and sampling sufficiency was calculated using EstimateS v.9 [54]. Data were transformed by square root to reduce the influence of common species and differences in total abundance, and a Bray-Curtis similarity matrix was constructed using communities from different fragments. SIMPER [54] was used to determine the contribution of each species to dung beetle community structure. Analysis of similarities -ANOSIM [55] was used to test differences between dung beetle communities.

The matrices of explanatory data were analyzed and ordinations were performed. A Principal Components analysis (PCA) of environmental variables was calculated using Primer [55] and Principal Coordinates Analysis (PCoA) of management variables was calculated using Hamann similarity in R 3.0.1 [56]. Analysis of similarities (ANOSIM) [55] was used to test differences between the environmental variables, as well as management variables. The mammal richness matrix was used without transformation. Spatial predictors were created using Principal Coordinates of Neighbour Matrices (PCNM) [57– 58], which is part of a set of spatial eigenfunction analyses called Moran’s Eigenvector Maps. The response variables of the dung beetles communities were species richness, abundance and biomass per fragment. The relation of the latter variables to predictor variables (such as vegetation (PCA1), management (PCoA1), mammalian richness, spatial distribution (PCNM1), fragments size and altitude) was initially observed in an exploratory analysis with multiple regressions.

Generalized Linear Mixed Models -GLMMs [59] with a Poisson error distribution [56], were used to test effects of each set of explanatory variables and combined effects of explanatory variables on dung beetle communities in the two types of fragments (conventional and transgenic). In GLMMs, type of maize was considered as a fixed factor. In all analyses performed, the fragment was used as the sampling unit.

### Results

#### Dung beetle communities

A total of 3454 dung beetles belonging to 44 species were collected. *Uroxys* aff. *terminalis*, *Dichotomius* aff. *sericeus* and *Onthophagus* aff. *tristis* were the most abundant species in both fragment types, and together the three species accounted for 60% of abundance in fragments near GM maize, and 48% abundance near conventional maize (S1 Table).

Forty-two species and 2312 individuals were collected in fragments surrounded by conventional maize, and species richness per fragment ranged between two to 21. Thirty-eight species and 1142 individuals were collected in fragments surrounded by GM maize, with six to 25 species per fragment. The number of species observed was at least 80% of the species richness values generated by Chao 1, Chao 2 and Jackknife 1 estimators, demonstrating sampling sufficiency (Table 1).

**Table 1:** Abundance, observed richness, richness estimators Chao 1, Chao 2 and Jackknife 1, mean biomass per fragment, and total biomass calculated for the communities of Scarabaeinae beetles in fragments adjacent to GM and conventional maize in Campos Novos, Santa Catarina state, Brazil.

Ecological measures of Scarabaeinae community	Fragments adjacent to GM maize	Fragments adjacent to conventional maize
Abundance (N)	1142	2312
Richness (S)	38	42
Variation of richness per fragment	2 to 21	6 to 25
Estimated richness		
Chao1	43.24	43.42
Chao 2	45.12	48.10
Jackknife 1	47.5	51.5
Average biomass per individual	0.086 g	0.130 g
Total biomass	76.16 g	114.71 g

In analyzing the dung beetle community similarity within the 40 Atlantic Forest fragments, significant differences were found between dung beetle communities in fragments near conventional and GM maize (ANOSIM r = 0.081; p = 0.024). The five species that most contributed to the dissimilarity between fragments types were: *U*. aff. *terminalis* (15.25%), *D*. aff. *sericeus* (8.29%), *O*. aff. *tristis* (6.86%), *C*. *rutilans cyanescens* (6.10%) and *C*. aff. *trinodosum* (5.43%), since they were most abundant in conventional fragments.

#### Explanatory variables

Environment–According to the Principal Component Analysis (PCA) of environmental variables, both fragment types are homogeneous, with no separation according to the adjacent crop characteristics, transgenic or conventional. Axis 1 (PCA1) represents the “understory” which explained 24.6% of data variation, and was influenced by shrub diameter, shrub height and tree distance. Axis 2 (PCA2) represents the “forest canopy” which explained 18% of data variation, and was influenced by tree height, shrub diameter and tree crown diameter.

However, according to the variation in environmental variables, the fragments are homogeneous (ANOSIM r = 0.12; p = 0.006), without separation by type of crop (GM or conventional maize).

Crop management–A range of management combinations were found, including the use of insecticides in GM crops (S2 Table). The use of insecticide in the region aimed to control mainly the fall armyworm *Spodoptera frugiperda* (Smith, 1797), and corn earworms *Helicoverpa zea* (Boddie, 1850) and *Helicoverpa armigera* (Hübner, 1805).

The insecticide B*t* was the most used in conventional crops (five), and diamide and neonicotinoid in transgenic crops (six). The herbicide atrazine was the most used for weed control in conventional crops (nine) and atrazine (14) followed by glyphosate (11) in transgenic crops. Cattle were released after the harvest in conventional crops (12) and eight transgenic crops (S2 Table).

However, according to the variation in management variables, the fragments are homogeneous (ANOSIM r = 0.27; p = 0.99), without separation by type of crop (GM or conventional maize).

Mammals–A total of 26 large and medium mammal species were found within the forest fragments, of which 21 are native mammals. The majority of species were found in both fragment types. A total of 19 mammal species were found in fragments surrounded by transgenic maize and 25 mammal species in fragments surrounded by conventional maize (S3 Table). Six different mammal species were ‘rare’ and had only one or two records each. The availability of mammalian dung was not assessed on these small fragments since most mammals are non-resident and visit the fragments opportunistically. The mammal richness ranged from 1 to 11 species per fragment (S4 Table). In many fragments mammal cubs were detected (i.e., *Procyon cancrivorus*, *Cerdocyon thous*, *Mazama gouazoubira*, *Nasua nasua*), revealing that the period before maize harvest coincides with mammal reproduction. The mammal richness was not correlated to fragment size and was also not correlated to dung beetle species richness and abundance.

Spatial configuration—The total study area including the forest fragments and crops was 790 km^2^, with a width of 24 km and length of 47 km. The distance between the fragments ranged from 14 m to 6.5 km and the two fragment types are randomly scattered in the area (Fig 1). PCNM analysis was carried out using a truncated distance matrix and eight statistically significant vectors were selected with the Moran index.

#### Relationship between dung beetles and environment

In a second evaluation, aiming to examine the set of variables that may have influenced dung beetle community, the first variables of each test (PCNM´s with spatial distances, PCA with environmental variables, PCoA with management variables) were extracted and multiple regressions were performed. Fragment size, altitude and mammal richness were also included as explanatory variables. Regarding species richness, multiple regressions showed that dung beetle species richness was related to fragment type, conventional or transgenic (t = 3.17, p = 0.003). Furthermore, dung beetle richness was positively correlated with fragment size (t = 4.76, p = 0.003), spatial distance (PCNM 1) (t = 5.48, p = 0.004), and management (PCoA1) (t = 2.00, p = 0.003), conversely, it was not correlated with mammal richness, environment (PCA1) and altitude. The abundance of dung beetles was also correlated to fragment type, conventional or transgenic (t = 2.24, p = 0.03), and to spatial distance (PCNM1) (t = 4.5, p = 0.007), and was not correlated to fragment size, management (PCoA1), environment (PCA1), mammal richness and altitude. The total biomass was correlated to fragment type, conventional or transgenic (t = -2.55, p = 0.015), it was positively correlated to fragment size (t = 5.26, p = 0.001), and it was negatively correlated with mammal richness (t = -2.07, p = 0.045), and it had no correlation with spatial distance (PCNM1), management (PCoA1), environment (PCA1) and altitude (S4 Table).

**Fig 2:**
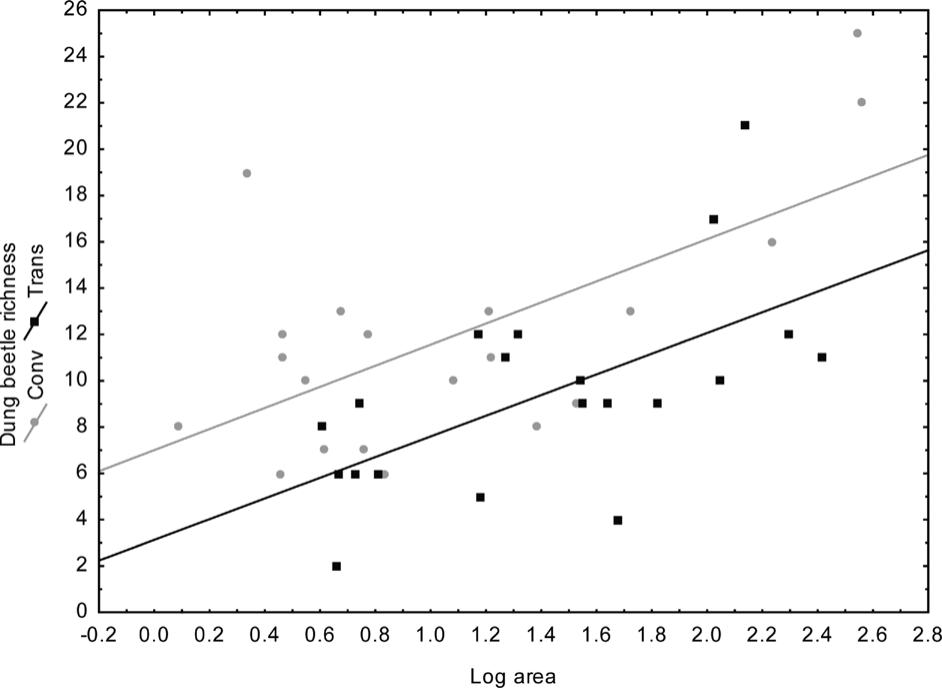
Dung beetle species richness in relation to fragment size (log area) in 40 Atlantic Forest fragments adjacent to transgenic and conventional maize in Campos Novos, Southern Brazil.

As predicted for fragmented areas, larger fragments had greater species richness (ANCOVA: R^2^ = 0.43), and dung beetle species richness was greater in fragments surrounded by conventional maize (F = 11; p = 0.002) (Fig 2).

**Fig 3:**
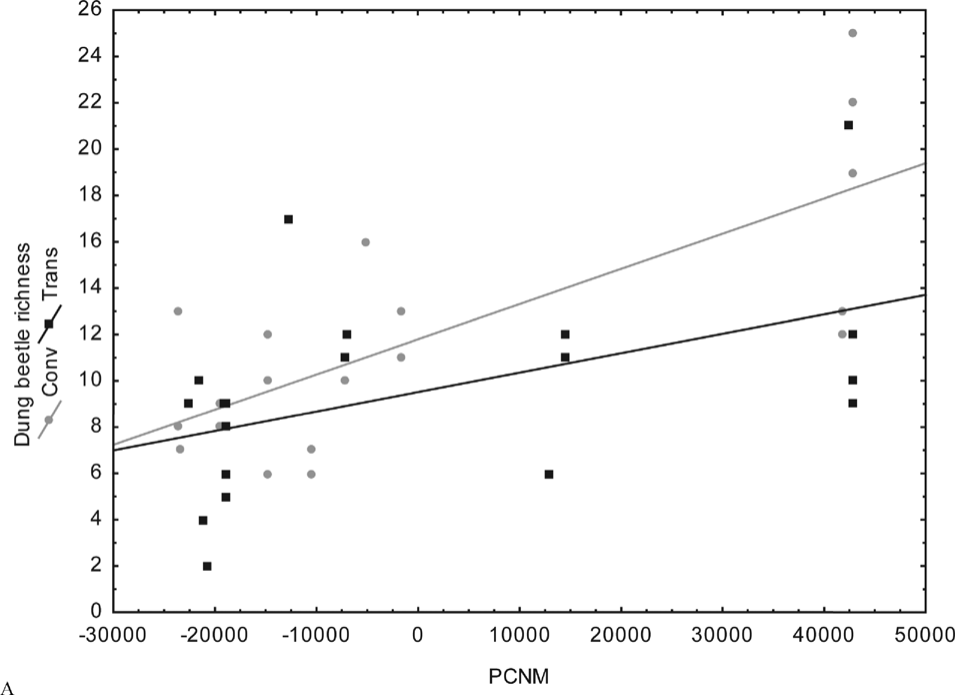
Distribution of dung beetle species richness in relation to spatial distance (PCNM) in 40 Atlantic Forest fragments adjacent to transgenic and conventional maize in Campos Novos, Southern Brazil.

**Fig 4:**
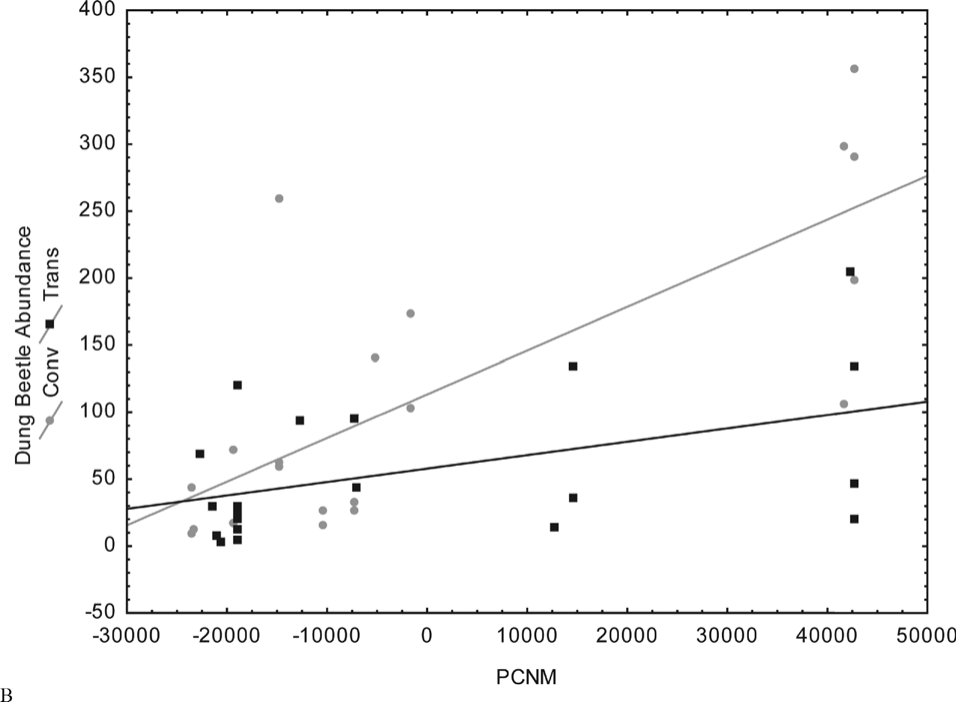
Distribution of dung beetle abundance in relation to spatial distance (PCNM) in 40 Atlantic Forest fragments adjacent to transgenic and conventional maize in Campos Novos, Southern Brazil.

Spatial distance (PCNM) was correlated with dung beetle species richness and abundance in both fragment types. Dung beetle species richness and abundance were higher in more distant fragments. However, dung beetle species richness and abundance in fragments surrounded by conventional maize were greater than dung beetle species richness and abundance in fragments surrounded by GM maize. The closest fragments showed similar species richness (Fig 3) and abundance (Fig 4).

When we tested the effects of each set of independent variables: vegetation (PCA), and spatial distance (PCNM) were important explanatory variables for dung beetle communities (Table 2). And the combined effects of variables: type of maize (conventional or transgenic) combined with the PCA (vegetation), type of maize combined with fragment area, type of maize combined with PCNM and type of maize combined with maize management also were important to dung beetle communities (Table 2). The set of variables of management was significant only associated with the factor (conventional or transgenic) (Table 2).

**Table 2:** GLMM’s results of explanatory variables of dung beetle communities in 20 fragments adjacent to transgenic and 20 fragments adjacent to conventional maize, in Campos Novos, Southern Brazil.

Effect	z value	Pr(>|z|)	AIC
Intercept	6.90	<0.001	1650.6
Factor (type of maize)	-1.40	0.160	
**PCA (vegetation)**	**5.16**	**<0.001**	
Fragment area	-0.65	0.513	
**PCNM**	8.86	**<0.001**	
Management	1.80	0.070	
PCA *factor	**-2.50**	**0.002**	
Fragment area *factor	**8.95**	**<0.001**	
PCNM *factor	**-1.16**	**<0.001**	
Management *factor	**6.03**	**<0.001**	

Significant effects are in bold.

### Discussion

Our results showed that both dung beetle community structure and composition are different in fragments surrounded by GM maize when compared with fragments surrounded by conventional maize, confirming previous findings, where were detected differences in the proportion of functional groups and abundance of some species of dung beetles in Atlantic forest fragments surrounded by GM maize [13]. In addition, dung beetle communities from fragments near GM maize showed lower species richness, total abundance and total biomass. These differences are attributed to maize management techniques and type of maize (conventional or transgenic) surrounding the forest fragment.

Dung beetle species richness at the landscape level reveals a great diversity even in a region with many Atlantic Forest fragments surrounded by a matrix composed of soybean and maize (see [60, 46]). Small forest fragments have been frequently referenced as habitats that are unsuitable for many animals, including large-bodied mammals and associated coprophagous beetles [61]. However, this study found mammal richness to be large in the region, and possibly these mammals use small fragments as stepping-stones or corridors to move to core areas. The majority of mammals registered in this study can disperse for many kilometers and this explains the similarity of mammals within the two fragment types. For example, although the puma (*Puma concolor*) was recorded in a small fragment (1.2 ha) there are larger fragments in the region. The distribution of dung beetles is strongly influenced by the diversity of mammal excrements [62–64]. Mammal diversity would explain the high species richness and abundance of dung beetles found in the region, since dung beetle community structure is based on resource availability (bottom up), and the spatial and temporal competition for resources is a strong modifier of dung beetle population dynamics [65].

Fragment size (area) was an important explanatory variable for the dung beetle communities within fragment types, where dung beetle richness and abundance was greater in fragments near conventional maize. It is well known that dung beetles are sensitive to habitat loss and fragmentation and a considerable number of species are forest-dependent [30, 34, 66– 67]. Furthermore, increased dung beetle species richness and abundance was correlated with spatial distance in both fragment types. Spatial limitation of dung beetles may be related to the geographic distance or lack of connectivity caused by fragmentation [46]. The dispersal abilities of different dung beetle species are poorly known, but some research shows that it may vary between 300 and 1500 m depending on the species and landscape [47–49, 68– 69]. It is interesting to note in this study the communities with greater richness and abundance were located in more distant forest fragments in the middle of conventional maize. Since the forest and consequently dung beetles are directly influenced by land use, we suggest that smaller fragments must be managed in order to maintain connected mosaics. Furthermore, the crops surrounding the fragments should be managed to minimize effects on forest fragments and improve connectivity.

Environmental variables influences dung beetle assemblages [34, 60, 70– 71], and the variables related to forest cover (tree and shrub height) shown as important effects on dung beetle variation. These variables are related to factors such as sunlight and humidity, which could affect dung beetle reproduction [72]. Environmental heterogeneity has greater importance at smaller scales [46] and the prevalence of environmental effects indicates species sorting [73]: a metacommunity model where there are strong environmental controls and efficient dispersal, which allows species to track environmental changes [74].

The maize management techniques associated with type of maize cultivated surrounding the forest fragments influences the dung beetles communities present in the fragments. The management effect, especially the variables ‘cattle presence’ and ‘insecticide use’, were important predictive variables for dung beetle community, and ‘insecticide’ was positively related. However, the study area is predominantly used for agriculture, and insecticide has been applied for many years on the crops, even though within study areas insecticide was not applied during the study’s duration, it was applied in previous years. The use of GM maize in these areas was an attempt to decrease application of insecticides (although in seven GM areas insecticide was applied); however the dung beetle community response to this disturbance was worst in terms of dung beetle richness and abundance than in fragments adjacent to conventional maize with insecticide. Thus, the remaining dung beetles species found in the forest fragments in this study are already possibly less affected by this disturbance.

Cattle presence and the indirect use of ivermectin negatively affect the composition and abundance of dung beetles in fragments surrounded by maize. The residuals of ivermectin are released in excrements, which contaminate the environment and can affect dung beetles [75–78]. Some species, unlike the majority, were benefited in fragments where cattle had open access (i.e., *U*. aff. *terminalis*), demonstrating which can be less affected.

Herbicide use was positively related with abundance and negatively with dung beetle biomass. There was an increase in smaller dung beetle species abundance with less biomass. Herbicides are applied in all GM maize crops, as well as most of the conventional maize crops, and herbicide application can cause a decline in the majority of dung beetles and impair reproductive function [79]. Forest-dependent dung beetle species depend in part on their ability to survive in human-modified landscapes [80].

Even in the absence of ecophysiological studies that may determine the effect of GM maize on dung beetle species and, consequently, the effects on dung beetle communities, in Southern Brazil’s scenario, where large fields of monocultures threaten biodiversity, the use of GM maize combined with associated agricultural management techniques may be accelerating the dung beetle loss in Atlantic Forest fragments adjacent to cornfields and, subsequently, the loss of ecosystem services provided by dung beetles.

## Supporting Information

S1 Table Scarabaeinae species collected in 40 fragments (February 2013 and 2014) of Atlantic Forest in the region of Campos Novos, Southern Brazil. T: fragments adjacent to GM maize, C: fragments adjacent to conventional maize.
(DOC)

S2 Table Crop management near the 40 forest fragments in Campos Novos, Santa Catarina, Brazil. C: fragments adjacent to conventional maize, T: fragments adjacent to transgenic maize.
(DOC)

S3 Table Mammal species records in 40 Atlantic Forest fragments surrounded by conventional maize (20) or transgenic maize (20) in Campos Novos, Southern Brazil. (DOC)

S4 Table Dung beetle community data, mammal richness, and measurements from 40 forest fragments in Campos Novos, Southern Brazil. First principal component (PCA1), first principal coordinates analysis of management (PCoA1) and first principal coordinates of neighbor matrices (PCNM1). C: fragments adjacent to conventional maize. T: fragments adjacent to transgenic maize.
(DOC)
