# Supplementary material for: The Importance of Maize Management on Dung Beetle Communities in Atlantic Forest Fragments
Source: PLoS One. 2015 Dec 22;10(12):e0145000. doi: 10.1371/journal.pone.0145000 (PMC4690589; doi:10.1371/journal.pone.0145000)
Supplement: S1 Table — T: fragments adjacent to GM maize, C: fragments adjacent to conventional maize. (DOC) [file pone.0145000.s001.doc]

**S1- Table 1**: Scarabaeinae species collected in 40 fragments (February 2013 and 2014) of Atlantic Forest in the region of Campos Novos, Southern Brazil. T: fragments adjacent to GM maize, C: fragments adjacent to conventional maize.

| Tribe / Species |  | | Total |
| --- | --- | --- | --- |
|  | T | C |  |
| Ateuchini |  |  |  |
| *Uroxys* aff. *terminalis* Waterhouse, 1891 | 261 | 912 | 1173 |
| *Uroxys* sp. | 27 | 51 | 78 |
| Coprini |  |  |  |
| *Canthidium cavifrons* Balthasar, 1939 | 13 | 12 | 25 |
| *Canthidium* aff. *dispar* Harold, 1867 | 9 | 8 | 17 |
| *Canthidium moestum* Harold, 1867 | 0 | 1 | 1 |
| *Canthidium* sp | 0 | 7 | 7 |
| *Canthidium* aff. *trinodosum* (Boheman, 1858) | 81 | 153 | 234 |
| *Canthidium* sp1 | 1 | 2 | 3 |
| *Dichotomius assifer* Eschscholtz, 1822 | 1 | 7 | 8 |
| *Dichotomius bicuspis* Germar, 1824 | 9 | 19 | 28 |
| *Dichotomius depressicollis* (Harold, 1867) | 0 | 1 | 1 |
| *Dichotomius fissus* (Harold, 1867) | 0 | 1 | 1 |
| *Dichotomius* aff. *punctulatipennis* (Luederwaldt, 1930) | 4 | 5 | 9 |
| *Dichotomius* aff. *pygidialis*  (Luederwaldt, 1922) | 10 | 2 | 12 |
| *Dichotomius* aff. *sericeus* (Harold, 1867) | 116 | 254 | 370 |
| *Dichotomius luctuosus* (Harold, 1869) | 1 | 1 | 2 |
| *Homocopris* sp. | 13 | 8 | 21 |
| *Ontherus azteca* Harold, 1869 | 4 | 42 | 46 |
| *Ontherus lobifrons* Génier, 1996 | 0 | 1 | 1 |
| *Ontherus sulcator* (Fabricius, 1775) | 1 | 6 | 7 |
| Deltochilini |  |  |  |
| *Canthon angularis* Harold, 1868 | 25 | 30 | 55 |
| *Canthon auricollis* Redtenbacher, 1867 | 13 | 3 | 16 |
| *Canthon ibarragrassoi* Martinez, 1952 | 6 | 16 | 22 |
| *Canthon* aff. *laminatus* Balthasar, 1939 | 2 | 0 | 2 |
| *Canthon lividus* *seminitens* Harold, 1868 | 47 | 45 | 92 |
| *Canthon luctuosus* Harold, 1868 | 17 | 18 | 35 |
| *Canthon* aff*. oliverioi* Pereira & Martínez, 1956 | 1 | 4 | 5 |
| *Canthon quadratus* Blanchard, 1846 | 1 | 3 | 4 |
| *Canthon rutilans cyanescens* Harold, 1868 | 108 | 145 | 253 |
| *Deltochilum brasiliense* (Castelnau, 1840) | 48 | 23 | 71 |
| *Deltochilum cristatum* Paulian, 1938 | 36 | 63 | 99 |
| *Deltochilum riehli* Harold, 1868 | 5 | 28 | 33 |
| *Deltochilum rubripenne* Gory, 1831 | 1 | 0 | 1 |
| Oniticellini |  |  |  |
| *Eurysternus aeneus* Génier, 2009 | 0 | 2 | 2 |
| *Eurysternus caribaeus* (Herbst, 1789) | 16 | 2 | 18 |
| *Eurysternus cyanescens* Balthasar, 1939 | 6 | 2 | 8 |
| *Eurysternus francinae* Génier, 2009 | 13 | 48 | 61 |
| *Eurysternus navajasi* Martinez, 1988 | 1 | 2 | 3 |
| *Eurysternus parallelus* Castelnau, 1840 | 35 | 107 | 142 |
| Onthophagini |  |  |  |
| *Onthophagus catharinensis* Paulian, 1936 | 22 | 3 | 25 |
| *Onthophagus* aff. *hirculus* Mannerheim, 1829 | 4 | 24 | 28 |
| *Onthophagus* aff. *tristis* Harold, 1873 | 168 | 230 | 398 |
| Phanaeini |  |  |  |
| *Coprophanaeus saphirinus* (Sturm, 1826) | 14 | 19 | 33 |
| *Sulcophanaeus menelas* (Castelnau, 1840) | 2 | 2 | 4 |
