# Supplementary material for: The Importance of Maize Management on Dung Beetle Communities in Atlantic Forest Fragments
Source: PLoS One. 2015 Dec 22;10(12):e0145000. doi: 10.1371/journal.pone.0145000 (PMC4690589; doi:10.1371/journal.pone.0145000)
Supplement: S2 Table — C: fragments adjacent to conventional maize, T: fragments adjacent to transgenic maize. (DOC) [file pone.0145000.s002.doc]

**S2- Table 2**: Crop management near the 40 forest fragments in Campos Novos, Santa Catarina, Brazil. C: fragments adjacent to conventional maize, T: fragments adjacent to transgenic maize.

|  | Maize variety | Gene  Toxin | Cattle | Inseticide | Herbicide | Fungicide | GM crop ever |
| --- | --- | --- | --- | --- | --- | --- | --- |
| C1 | Colorado |  | - | Deltametrina | Atrazine | - | - |
| C2 | Colorado |  | - | Deltametrina | Atrazine | - | - |
| C3 | Guerra |  | X | - | Glyfosate-Callisto | - | - |
| C4 | Guerra |  | X | - | Glyfosate-Callisto | - | - |
| C5 | Guerra |  | X | - | Glyfosate-Callisto | - | - |
| C6 | Aztec |  | - | - | - | - | - |
| C7 | Pixirum |  | X | - | - | - | - |
| C8 | Pixirum |  | X | - | - | - | - |
| C9 | Pixirum |  | X | - | - | - | - |
| C10 | Pixirum |  | X | - | - | - | - |
| C11 | AG 8021 |  | X | Diamida | Atrazine- Soberan | - | X |
| C12 | Dow 2B587 |  | - | Bt | Atrazine- Soberan-Simazine | - | X |
| C13 | AS 1570 |  | - | Bt | Atrazine- Soberan-Simazine | - | X |
| C14 | P30F53 |  | X | Bt | Atrazine- Soberan-Simazine | - | X |
| C15 | P2530 |  | - | Bt | Atrazine- Soberan-Simazine | - | X |
| C16 | AG 8021 |  | X | Diamida | Atrazine- Soberan | - | X |
| C17 | AS 1551 |  | - | Bt | Atrazine- Soberan-Simazine | - | X |
| C18 | Pixirum |  | - | - | 0 | - | X |
| C19 | Guerra SG 6302 |  | X | - | 0 | - | - |
| C20 | Guerra SG 6302 |  | X | - | Gramoxone | - | - |
| T1 | P1630H | Cry1F | - | - | Tembotrione-Atrazine | - | X |
| T2 | P1630H | Cry1F | - | - | Tembotrione- Atrazine | - | X |
| T3 | Defender/Maximus | Cry1Ab | X | - | Zapp + Glyphosate Graminicide | - | X |
| T4 | Defender/Maximus | Cry1Ab | X | - | Zapp + Glyphosate Graminicide | - | x |
| T5 | Defender/Maximus | Cry1Ab | X | - | Zapp + Glyphosate Graminicide | - | x |
| T6 | DKB 250Pro | Cry1A.105 | - | - | Glyphosate Atrazine | Strobilurin - Triazole | x |
| T7 | DKB 250Pro | Cry1A.105 | - | - | Glyphosate Atrazine | Strobilurin - Triazole | x |
| T8 | DKB 250Pro | Cry1A.105 | - | - | Glyphosate Atrazine | Strobilurin - Triazole | x |
| T9 | DKB 250Pro | Cry1A.105 | - | - | Glyphosate Atrazine | Strobilurin - Triazole | x |
| T10 | DKB 250Pro | Cry1A.105 | - | - | Glyphosate Atrazine | Strobilurin - Triazole | x |
| T11 | AG 8021 YG | Cry1Ab | X | Diamida | Atrazina- Soberan | - | x |
| T12 | AG 8021 YG | Cry1Ab | X | Diamida | Atrazina- Soberan | - | x |
| T13 | Dow 2B587Hx | Cry1F | - | Bt | Atrazina- Soberan-Simazine | - | x |
| T14 | DKB 240Pro2 | Cry1A.105 | - | Imidacloprid | Atrazine- Glyphosate | Strobilurin-Triazole | x |
| T15 | DKB 240Pro2 | Cry1A.105 | - | Imidacloprid | Atrazine- Glyphosate | Strobilurin - Triazole | x |
| T16 | DKB 240Pro2 | Cry1A.105 | - | Imidacloprid | Atrazine- Glyphosate | Strobilurin - Triazole | x |
| T17 | DKB 240 Pro | Cry1A.105 | X | - | Callisto | - | x |
| T18 | DKB 240Pro | Cry1A.105 | X | - | Callisto | - | x |
| T19 | DKB 240Pro | Cry1A.105 | X | - | Callisto | - | x |
| T20 | AG 8021 YG | Cry1Ab | - | Diamida | Atrazine- Soberan | - | x |
