# Supplementary material for: The Importance of Maize Management on Dung Beetle Communities in Atlantic Forest Fragments
Source: PLoS One. 2015 Dec 22;10(12):e0145000. doi: 10.1371/journal.pone.0145000 (PMC4690589; doi:10.1371/journal.pone.0145000)
Supplement: S3 Table — (DOC) [file pone.0145000.s003.doc]

**S3- Table 3**: Mammal species records in 40 Atlantic Forest fragments surrounded by conventional maize (20) or transgenic maize (20) in Campos Novos, Southern Brazil.

| Mammal species | Number of fragments with records in 20 fragments surrounded by | |
| --- | --- | --- |
|  | Conventional maize | Transgenic maize |
| *Mazama gouazoubira* (Fischer, 1814) | 14 | 20 |
| *Dasypus novemcinctus* Linnaeus, 1758 | 14 | 14 |
| *Cerdocyon thous* (Linnaeus, 1766) | 11 | 12 |
| *Didelphis albiventris* Lund, 1840 | 12 | 9 |
| *Nasua nasua* (Linnaeus, 1766) | 8 | 11 |
| *Dasypus septemcinctus* Linnaeus, 1758 | 9 | 2 |
| *Leopardus guttulus* (Hensel, 1872) | 5 | 6 |
| *Homo sapiens* Linnaeus, 1758 | 4 | 6 |
| *Euphractus sexcinctus* (Linnaeus, 1758) | 5 | 4 |
| *Bos taurus* Linnaeus, 1758 | 5 | 4 |
| *Canis lupus familiaris* Linnaeus, 1758 | 4 | 4 |
| *Procyon cancrivorus* (Cuvier, 1798) | 1 | 4 |
| *Dasyprocta azarae* Lichtenstein, 1823 | 2 | 3 |
| *Galictis cuja* (Molina, 1782) | 2 | 2 |
| *Eira barbara* (Linnaeus, 1758) | 1 | 2 |
| *Puma yagouaroundi* (Saint-Hilaire, 1803) | 1 | 2 |
| *Lepus europaeus* Pallas, 1778 | 2 | 1 |
| *Tamandua tetradactyla* (Linnaeus, 1758) | 1 | 2 |
| *Leopardus wiedii* (Schinz, 1821) | 2 | 0 |
| *Cuniculus paca (*Linnaeus, 1766) | 0 | 2 |
| *Conepatus chinga* (Molina, 1782) | 1 | 0 |
| *Sapajus nigritus* (Goldfuss, 1809) | 1 | 0 |
| *Felis catus* Linnaeus, 1758 | 1 | 0 |
| *Leopardus pardalis* (Linnaeus, 1758) | 1 | 0 |
| *Puma concolor* (Linnaeus, 1771) | 1 | 0 |
| *Hidrochaeris hidrochaeris* (Linnaeus, 1766) | 1 | 0 |
| Number of species | 25 | 19 |
