# Supplementary material for: The Importance of Maize Management on Dung Beetle Communities in Atlantic Forest Fragments
Source: PLoS One. 2015 Dec 22;10(12):e0145000. doi: 10.1371/journal.pone.0145000 (PMC4690589; doi:10.1371/journal.pone.0145000)
Supplement: S4 Table — First principal component (PCA1), first principal coordinates analysis of management (PCoA1) and first principal coordinates of neighbor matrices (PCNM1). C: fragments adjacent to conventional maize. T: fragments adjacent to transgenic maize. (DOC) [file pone.0145000.s004.doc]

**S4- Table 4: Dung beetle community data, mammal richness, and measurements from 40 forest fragments in Campos Novos, Southern Brazil. First principal component (PCA1), first principal coordinates analysis of management (PCoA1) and first principal coordinates of neighbor matrices (PCNM1). C: fragments adjacent to conventional maize. T: fragments adjacent to transgenic maize.**

|  | Year  of sampling | Dung beetle richness | Dung beetle abundance | Mammal richness | Fragment  area (m^2^) | Altitude (amsl) | Environment  PCA 1 | Management  PCoA1 | Spatial  PCNM 1 |
| --- | --- | --- | --- | --- | --- | --- | --- | --- | --- |
| C1 | 2013 | 13 | 44 | 3 | 16.338 | 932 | -0.36 | 3.20 | -23553 |
| C2 | 2013 | 8 | 10 | 6 | 24.379 | 950 | -2.07 | -3.20 | -23539 |
| C3 | 2013 | 10 | 60 | 8 | 3.522 | 826 | 0.84 | -6.60 | -14742 |
| C4 | 2013 | 12 | 260 | 8 | 5.949 | 888 | 0.06 | 3.26 | -14862 |
| C5 | 2013 | 6 | 63 | 6 | 6.827 | 900 | 0.98 | -1.55 | -14849 |
| C6 | 2013 | 7 | 13 | 6 | 4.136 | 915 | -2.85 | -1.96 | -23400 |
| C7 | 2013 | 8 | 18 | 2 | 1.216 | 861 | -0.08 | 7.42 | -19461 |
| C8 | 2013 | 9 | 72 | 7 | 33.681 | 853 | -0.69 | 9.25 | -19447 |
| C9 | 2013 | 6 | 27 | 3 | 2.870 | 847 | 1.73 | 7.28 | -10405 |
| C10 | 2013 | 7 | 16 | 4 | 5.718 | 833 | -0.30 | -1.11 | -10408 |
| C11 | 2014 | 10 | 33 | 11 | 12.006 | 909 | 2.56 | 9.46 | -7181 |
| C12 | 2014 | 13 | 107 | 5 | 53.216 | 786 | -0.69 | -1.31 | 41764 |
| C13 | 2014 | 12 | 299 | 6 | 2.897 | 751 | 1.45 | 1.48 | 41771 |
| C14 | 2014 | 25 | 357 | 8 | 347.615 | 784 | 1.16 | 7.90 | 42791 |
| C15 | 2014 | 19 | 290 | 5 | 2.156 | 817 | 1.99 | -1.35 | 42797 |
| C16 | 2014 | 11 | 27 | 4 | 16.405 | 910 | 2.76 | 8.06 | -7179 |
| C17 | 2014 | 22 | 199 | 5 | 364.771 | 773 | 1.49 | -1.43 | 42798 |
| C18 | 2014 | 13 | 103 | 5 | 4.725 | 793 | 0.95 | 3.69 | -1671 |
| C19 | 2014 | 11 | 174 | 5 | 2.927 | 768 | 2.05 | -3.72 | -1705 |
| C20 | 2014 | 16 | 140 | 2 | 172.382 | 753 | 0.93 | 4.89 | -5190 |
| T1 | 2013 | 10 | 29 | 9 | 111.591 | 915 | 0.44 | 2.14 | -21545 |
| T2 | 2013 | 9 | 68 | 7 | 35.634 | 944 | -1.62 | -2.14 | -22637 |
| T3 | 2013 | 6 | 121 | 8 | 5.332 | 878 | 1.44 | 9.71 | -18952 |
| T4 | 2013 | 8 | 23 | 5 | 4.016 | 893 | -2.63 | 8.71 | -18973 |
| T5 | 2013 | 9 | 29 | 9 | 65.818 | 890 | -2.97 | 4.76 | -18985 |
| T6 | 2013 | 6 | 12 | 6 | 6.517 | 894 | -2.61 | -1.52 | -18939 |
| T7 | 2013 | 5 | 5 | 6 | 15.242 | 891 | 3.39 | 2.65 | -18983 |
| T8 | 2013 | 9 | 20 | 6 | 43.672 | 873 | 0.30 | 2.65 | -19022 |
| T9 | 2013 | 2 | 3 | 6 | 4.562 | 891 | -4.09 | -2.28 | -20665 |
| T10 | 2013 | 4 | 8 | 4 | 47.312 | 896 | -2.31 | -1.49 | -21078 |
| T11 | 2014 | 11 | 96 | 2 | 260.237 | 912 | 0.18 | -1.27 | -7192 |
| T12 | 2014 | 12 | 44 | 6 | 14.918 | 853 | 0.42 | -1.27 | -7069 |
| T13 | 2014 | 21 | 205 | 5 | 18.676 | 828 | -0.15 | 9.50 | 42360 |
| T14 | 2014 | 6 | 14 | 5 | 136.743 | 911 | 1.20 | -6.57 | 12821 |
| T15 | 2014 | 11 | 36 | 1 | 4.611 | 917 | -1.52 | -5.82 | 14502 |
| T16 | 2014 | 12 | 134 | 5 | 196.514 | 953 | 0.30 | -6.73 | 14501 |
| T17 | 2014 | 12 | 20 | 7 | 20.764 | 767 | 1.25 | -4.44 | 42778 |
| T18 | 2014 | 10 | 47 | 6 | 34.505 | 782 | 0.41 | -6.24 | 42784 |
| T19 | 2014 | 9 | 134 | 2 | 5.554 | 739 | -0.16 | -1.25 | 42763 |
| T20 | 2014 | 17 | 94 | 5 | 106.167 | 872 | -3.15 | 3.00 | -12792 |
